# Supplementary figures and images for: Typhoid toxin sorting and exocytic transport from Salmonella Typhi-infected cells
Source: eLife. 2022 May 17;11:e78561. doi: 10.7554/eLife.78561 (PMC9142146; doi:10.7554/eLife.78561)

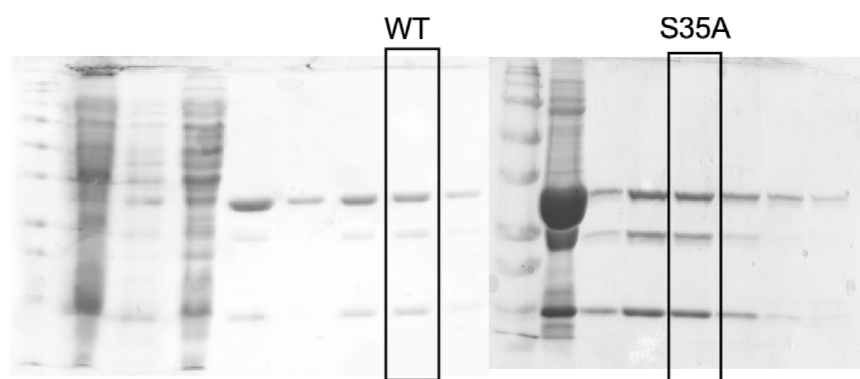

Supplement: Figure 1—source data 1. [file elife-78561-fig1-data1.pdf]

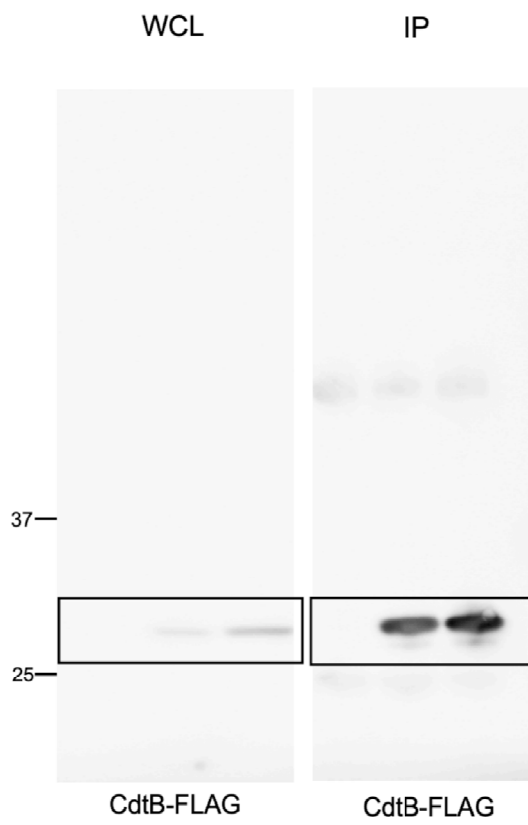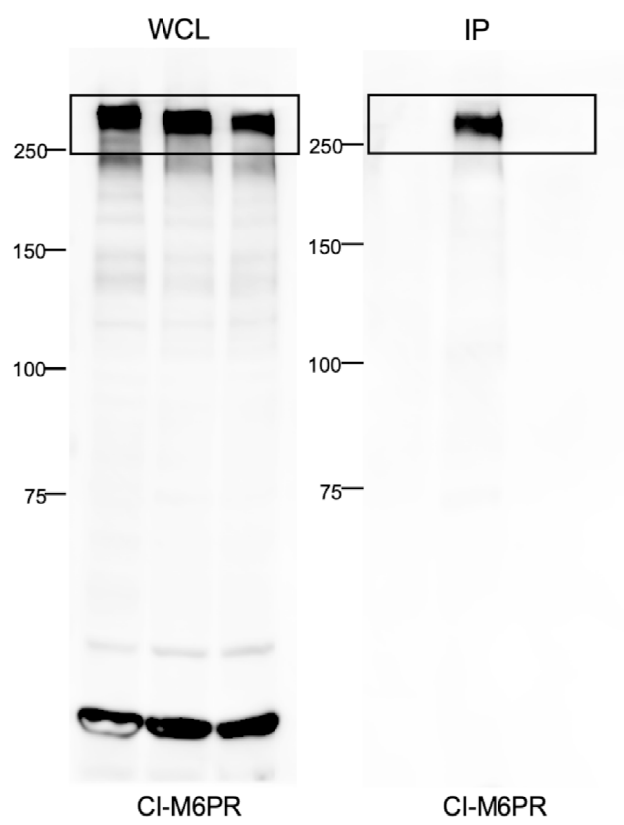

Supplement: Figure 1—source data 3. [file elife-78561-fig1-data3.pdf]

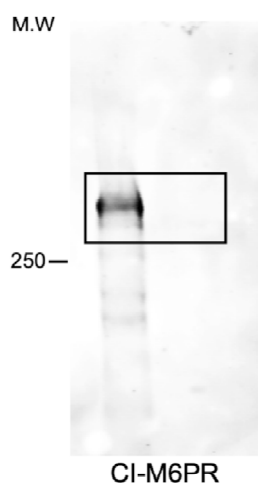

Supplement: Figure 2—source data 1. [file elife-78561-fig2-data1.pdf]

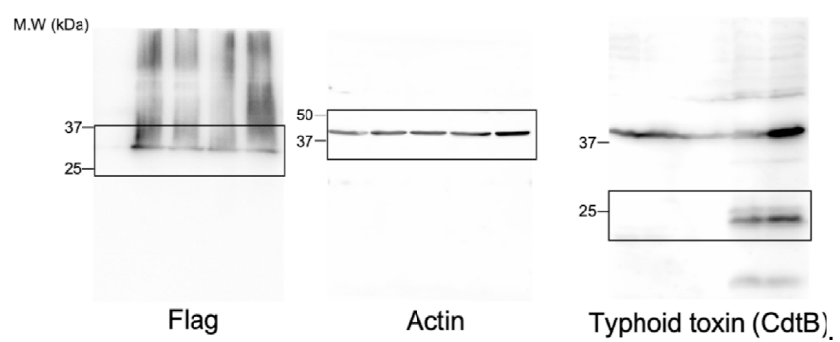

Supplement: Figure 2—figure supplement 3—source data 1. [file elife-78561-fig2-figsupp3-data1.pdf]

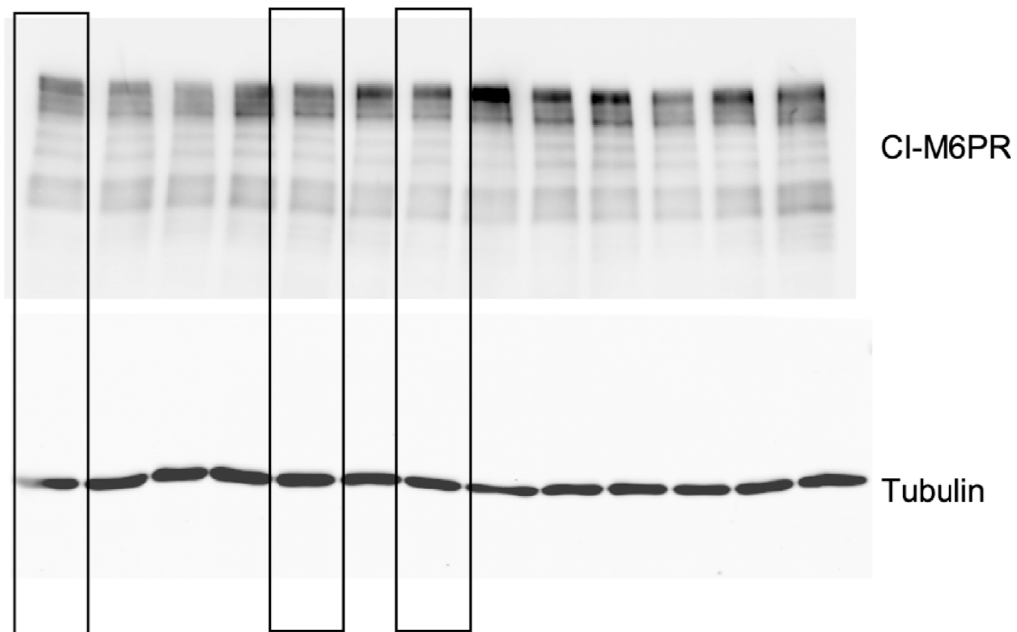

Supplement: Figure 3—figure supplement 6—source data 1. [file elife-78561-fig3-figsupp6-data1.pdf]

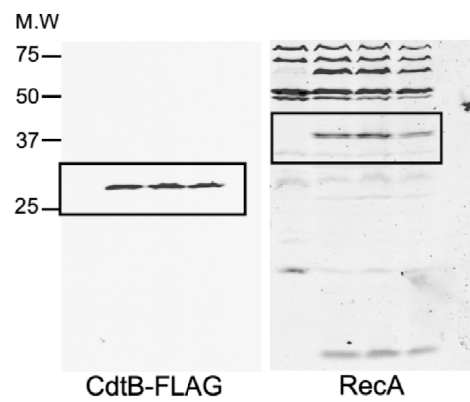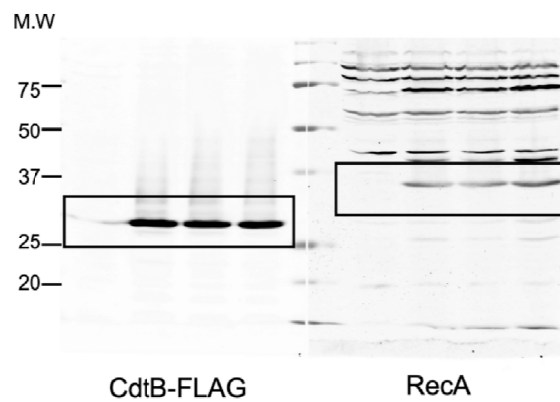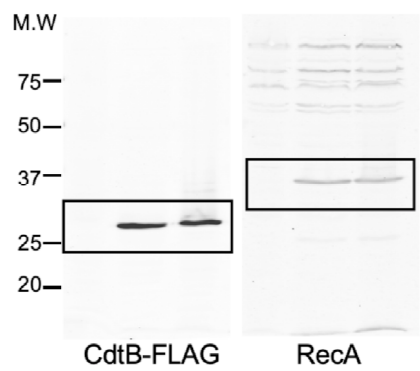

Supplement: Figure 4—source data 1. [file elife-78561-fig4-data1.pdf]

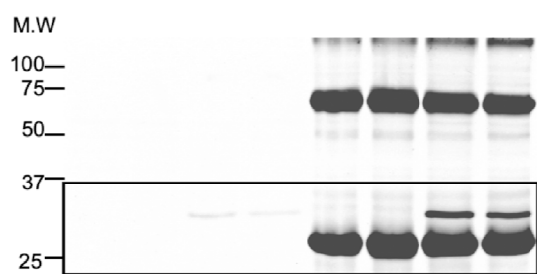

CdtB-FLAG

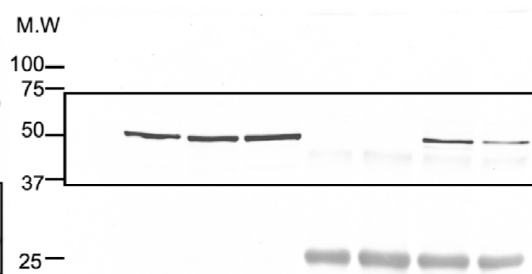

Sec23

Supplement: Figure 4—source data 3. [file elife-78561-fig4-data3.pdf]

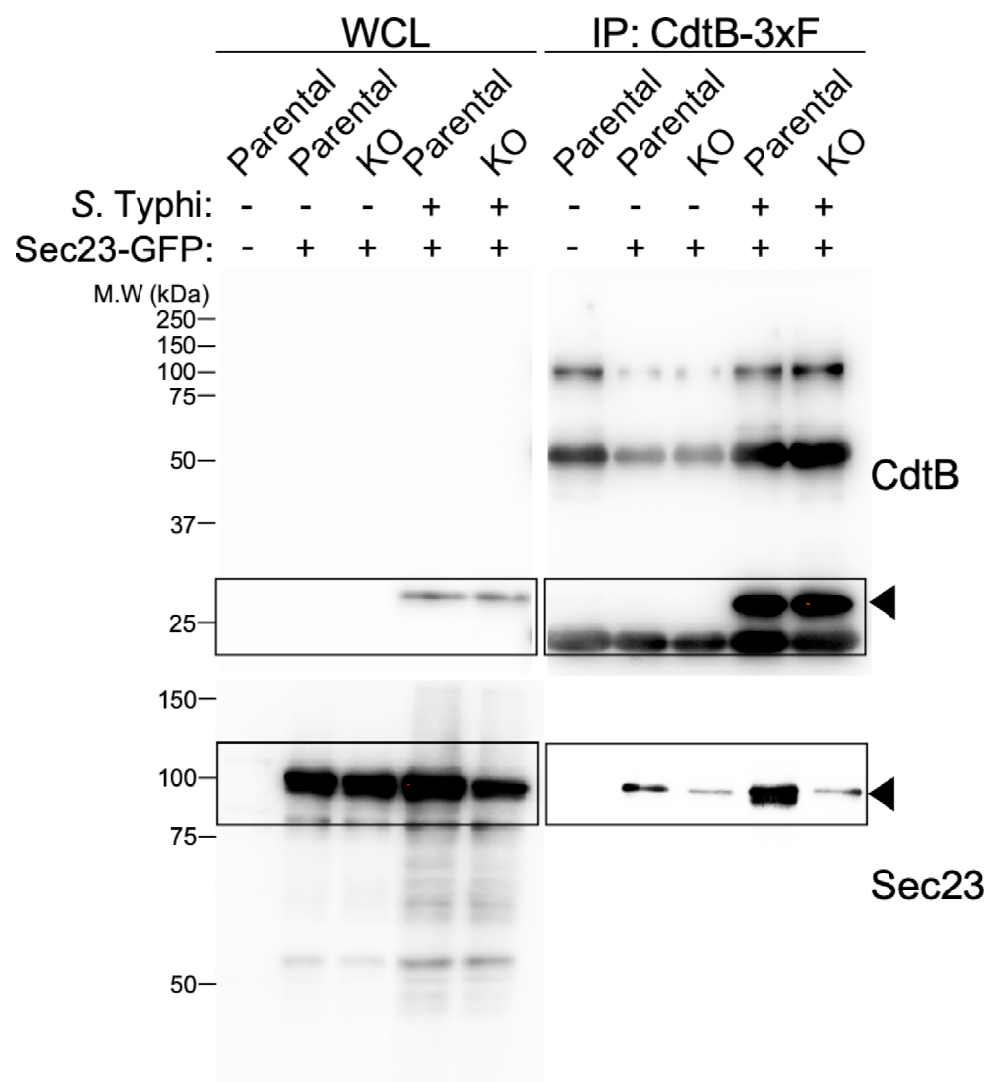

Supplement: Figure 4—source data 4. [file elife-78561-fig4-data4.pdf]

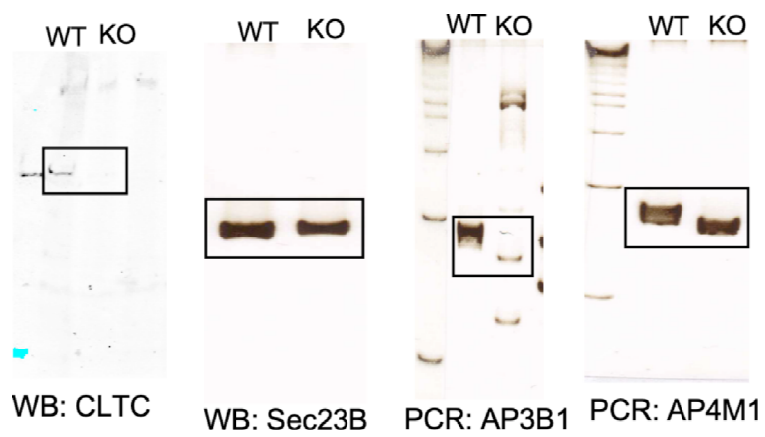

Supplement: Figure 4—figure supplement 1—source data 1. [file elife-78561-fig4-figsupp1-data1.pdf]

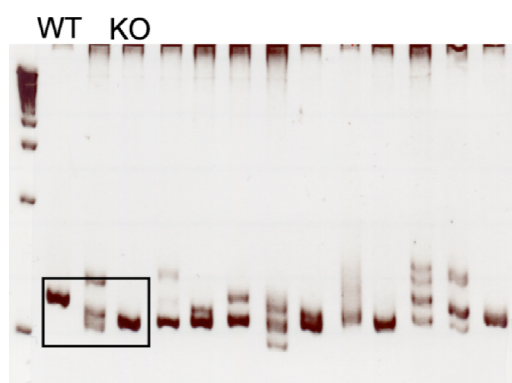

Supplement: Figure 4—figure supplement 1—source data 2. [file elife-78561-fig4-figsupp1-data2.pdf]

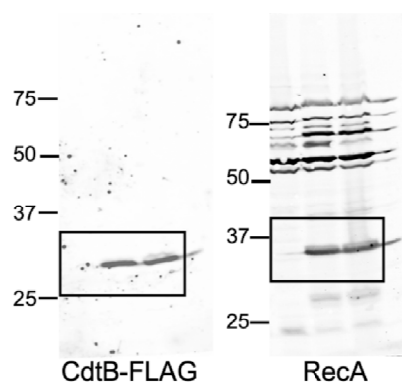

Supplement: Figure 5—source data 2. [file elife-78561-fig5-data2.pdf]

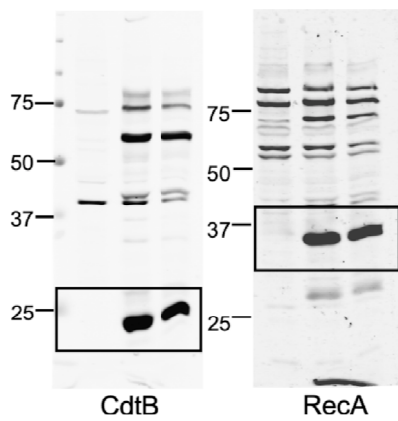

Supplement: Figure 5—source data 3. [file elife-78561-fig5-data3.pdf]

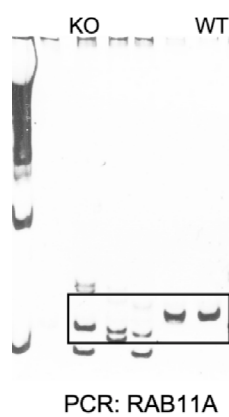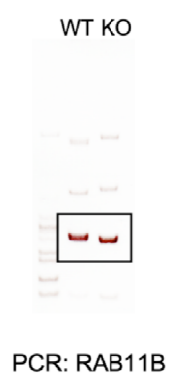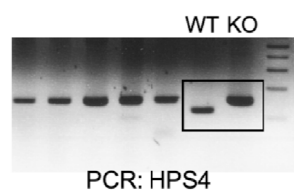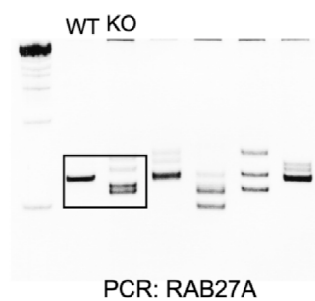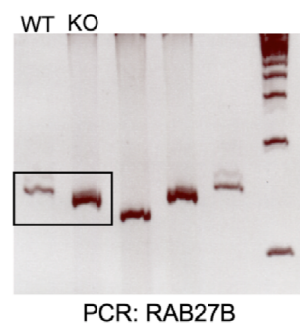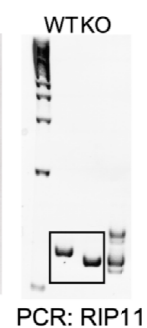

Supplement: Figure 5—figure supplement 1—source data 1. [file elife-78561-fig5-figsupp1-data1.pdf]

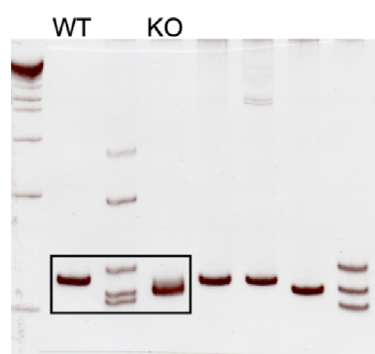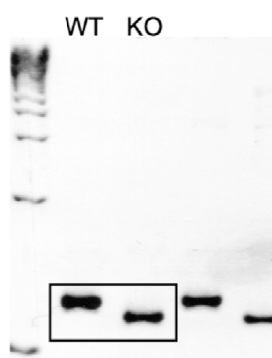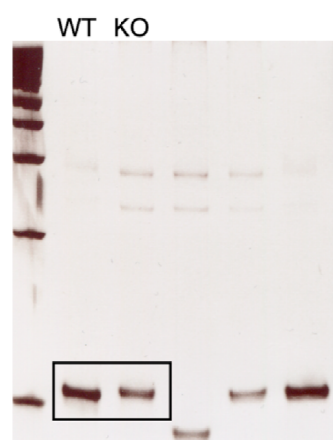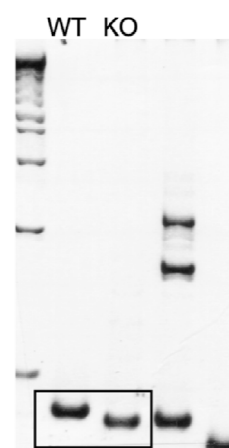

Supplement: Figure 5—figure supplement 1—source data 2. [file elife-78561-fig5-figsupp1-data2.pdf]

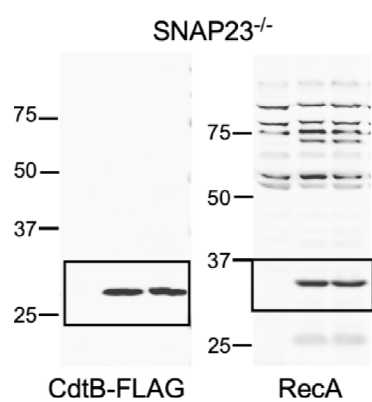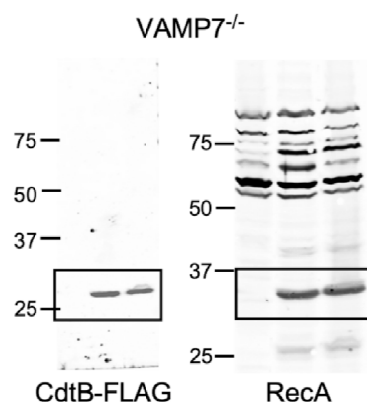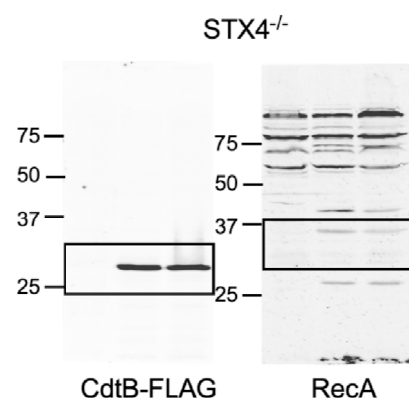

Supplement: Figure 6—source data 2. [file elife-78561-fig6-data2.pdf]
