# Supplementary material for: Typhoid toxin sorting and exocytic transport from Salmonella Typhi-infected cells
Source: eLife. 2022 May 17;11:e78561. doi: 10.7554/eLife.78561 (PMC9142146; doi:10.7554/eLife.78561)
Supplement: Supplementary file 1. [file elife-78561-supp1.docx]

**Supplementary File 1. List of strains used in this study.**

| **Strain** | **Genotype** |
| --- | --- |
| *Salmonella* Typhi: ISP2825 | wild type |
| SB300 | *S*. Typhipmurium SL1344 |
| SB2307 | *S.*Typhimurium-CdtB-3XFLAG (*STY1886, cdtB, ttsA, pltA, pltB*) |
| SB1946 | *S*. Typhi, CdtB-3XFLAG |
| SB3019 | *S*. Typhi, CdtB-3XFLAG, Δ*spiA* |
