# Supplementary material for: Typhoid toxin sorting and exocytic transport from Salmonella Typhi-infected cells
Source: eLife. 2022 May 17;11:e78561. doi: 10.7554/eLife.78561 (PMC9142146; doi:10.7554/eLife.78561)
Supplement: Supplementary file 2. [file elife-78561-supp2.docx]

**Supplementary File 2. List of plasmids used in this study.**

| **Plasmid** | **Description** |
| --- | --- |
| pSB5496 | pET28a-pltA-pltB-cdtB-3xflag-His |
| pSB5497 | pET28a-pltA-pltB S35A-cdtB-3xflag-His |
| pSB5849 | px459-sg-CI-M6PR (5'-CGGACTGAAGCTGGTGCGCA-3') |
| pSB5850 | px459-sg-Sec23B (5'-TACAATTGAGTACGTGATAC-3') |
| pSB5986 | px459-sg-Sar1B (5'-AATGTGCCTATACTGATTCT-3') |
| pSB5987 | px459-sg-CLTC (5'-TCGTTTTCAGGAGCATCTCC-3') |
| pSB5988 | px459-sg-AP3B1(5'-AAAGAAGAAGCCGTATACTA-3') |
| pSB5989 | px459-sg-AP4M1(5'-CTCTTTGACCTCAGCAGCGT-3') |
| pSB5990 | px459-sg-Rab11A (5'-CATTTCGAGTAAATCGAGAC-3') |
| pSB5991 | px459-sg-Rab11B (5'-GAGCAAGAGCACCATCGGCG-3') |
| pSB5992 | px459-sg-HPS4 (5'-GAAGGCGATCCAACAAGAGC-3') |
| pSB5993 | px459-sg-Rab27A (5'-AGTGGCTCCATCCGGCCCAC-3') |
| pSB5994 | px459-sg-Rab27B (5'-TCCTGGCCCTCGGGGATTCA-3') |
| pSB5995 | px459-sg-Rab11FIP5 (5'-GGTCAAACATACTGGCGCTC-3') |
| pSB5998 | px459-sg-SNAP23 (5'-AAGACAACATGGGGAGATGG-3') |
| pSB6001 | px459-sg--STX4 (5'-TGGTGCACCCGGGCACGGCA-3') |
| pSB6002 | px459-sg-EXOC7 (5'-TGACGAAGGCACTGACGCAG-3') |
| pSB6301 | px459-sg-VAMP7 (5'-TTCTGAATGAGATAAAGAAG-3') |
| pSB4136 | pWSKlacZ-GtgE |
| pSB4830 | pWSKlacZ-SopD2 |
| pSB5341 | pWSKlacZ-GtgA |
| pSB5342 | pWSKlacZ-SteB |
| pSB5343 | pWSKlacZ-SlrP |
| pSB5344 | pWSKlacZ-SspH1 |
| pSB5345 | pWSKlacZ-GogB |
| pSB5346 | pWSKlacZ-SseI |
| pSB5347 | pWSKlacZ-SseJ |
| pSB5348 | pWSKlacZ-SseK1 |
| pSB5349 | pWSKlacZ-SseK2 |
| pSB5350 | pWSKlacZ-SseK3 |
| pSB5581 | pWSKlacZ-SpvC |
| pSB5586 | pWSKlacZ-SpvD |
