# Supplementary material for: Typhoid toxin sorting and exocytic transport from Salmonella Typhi-infected cells
Source: eLife. 2022 May 17;11:e78561. doi: 10.7554/eLife.78561 (PMC9142146; doi:10.7554/eLife.78561)

**Figure 1- source data 1**

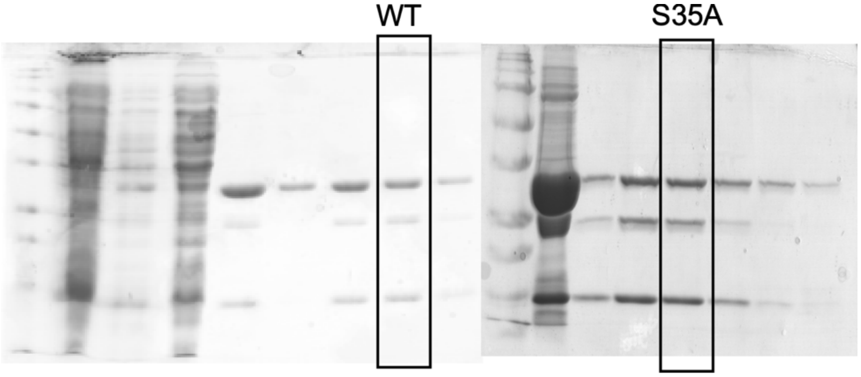

**Figure 1- source data 2**

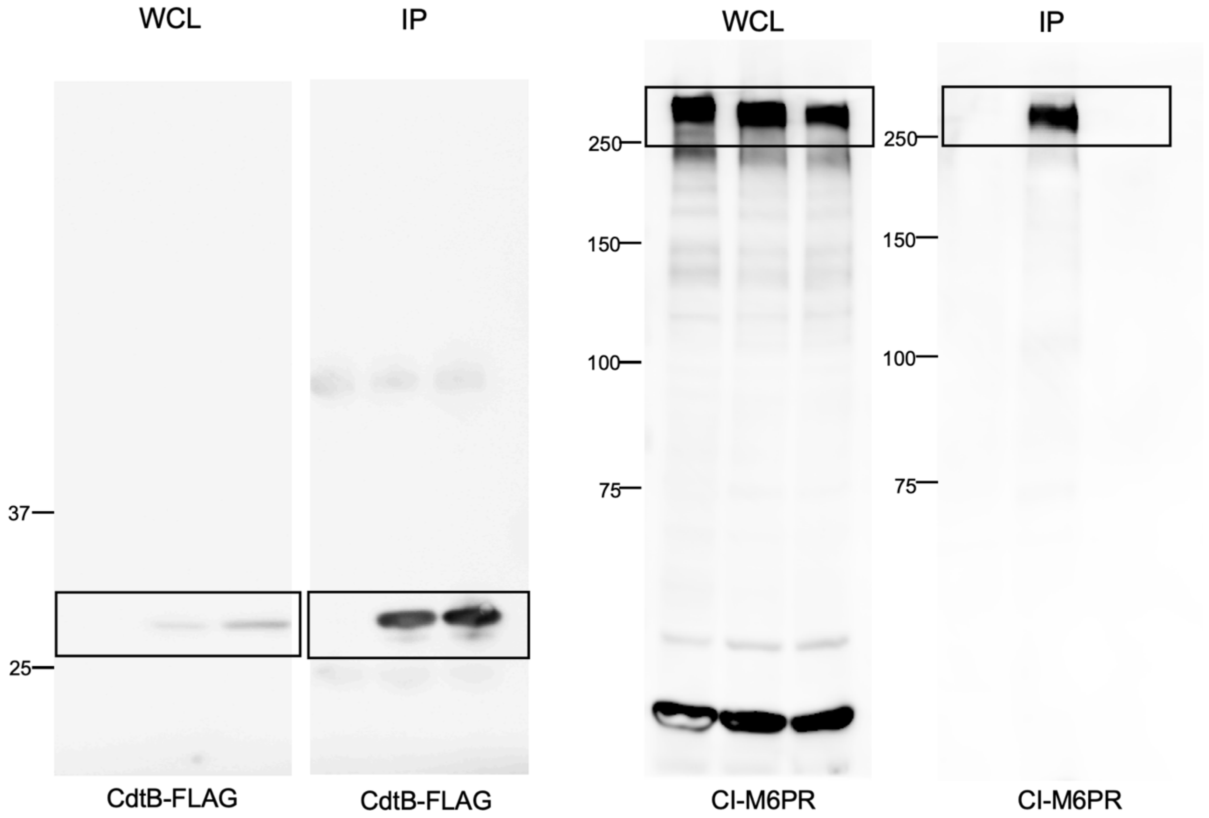

**Figure 2- figure supplement 4- source data 2**

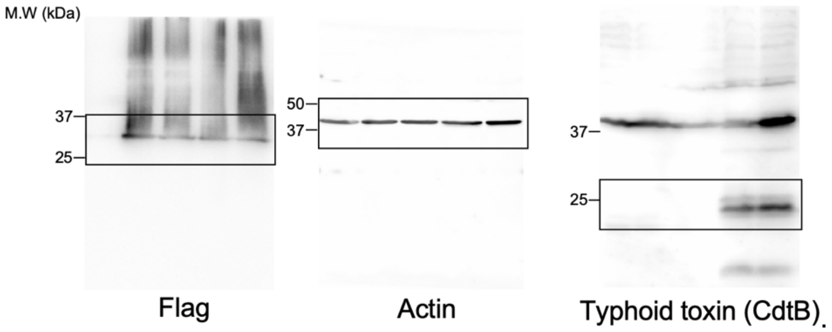

**Figure 2- source data 1**

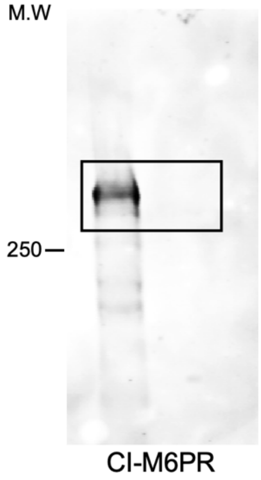

**Figure 3- figure supplement 10- source data 1**

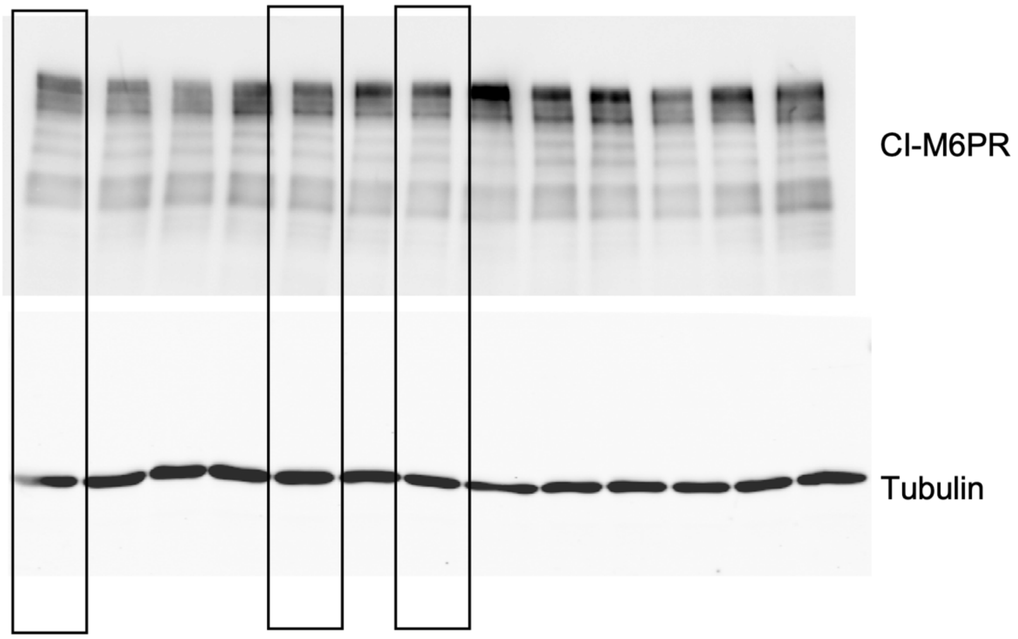

**Figure 4- figure supplement 3- source data 1**

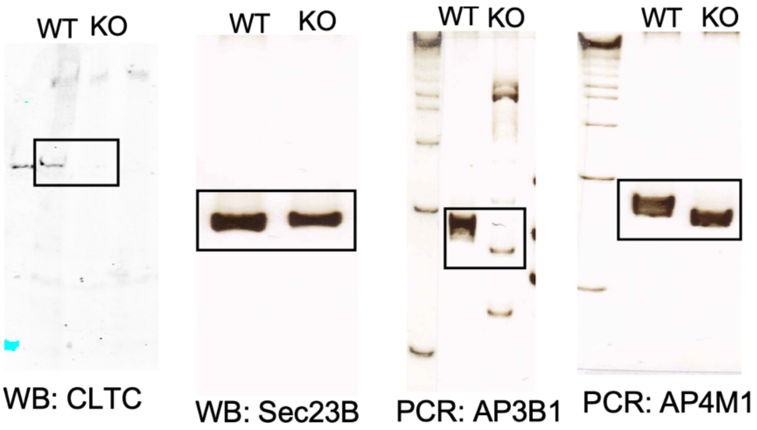

**Figure 4- figure supplement 3- source data 2**

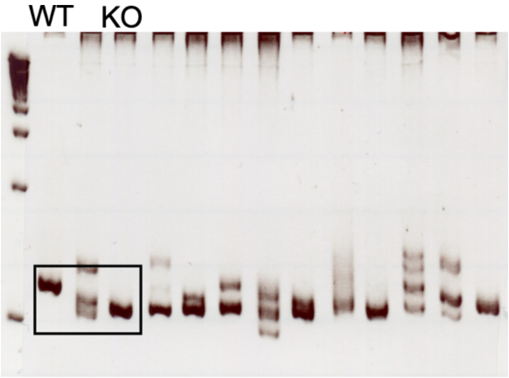


PCR: Sar1B

**Figure 4- source data 1**

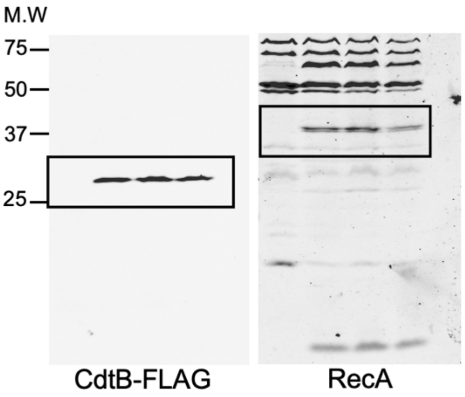


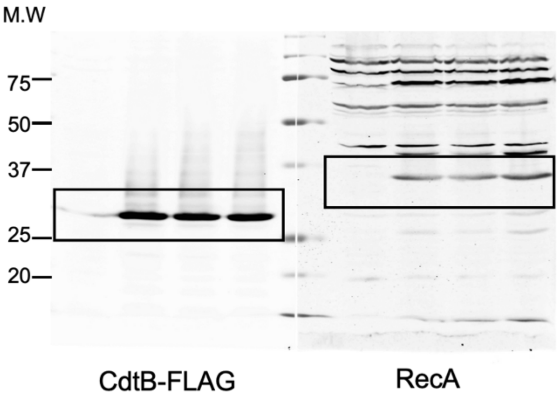


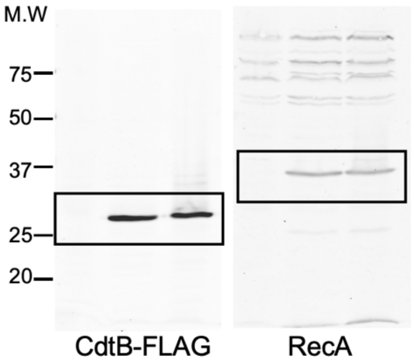

**Figure 4- source data 2**

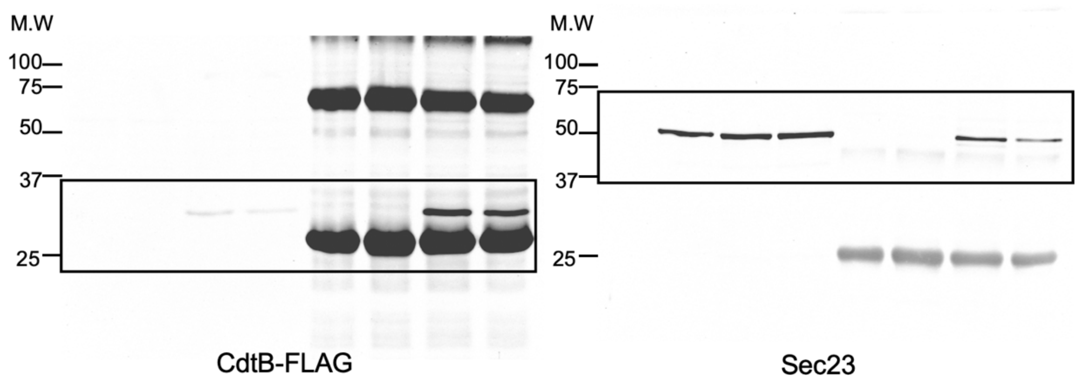

**Figure 4- source data 3**

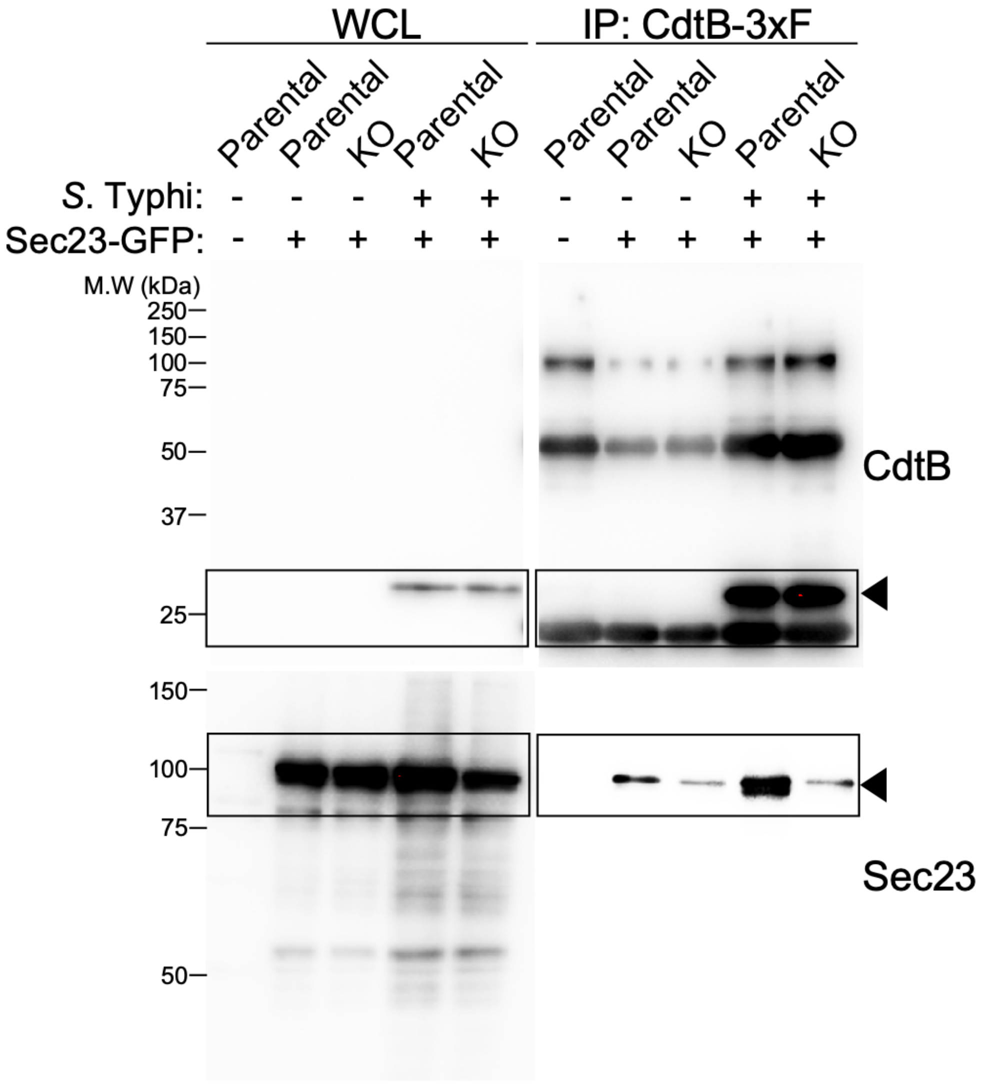

## Figure 5- figure supplement 6- source data 1

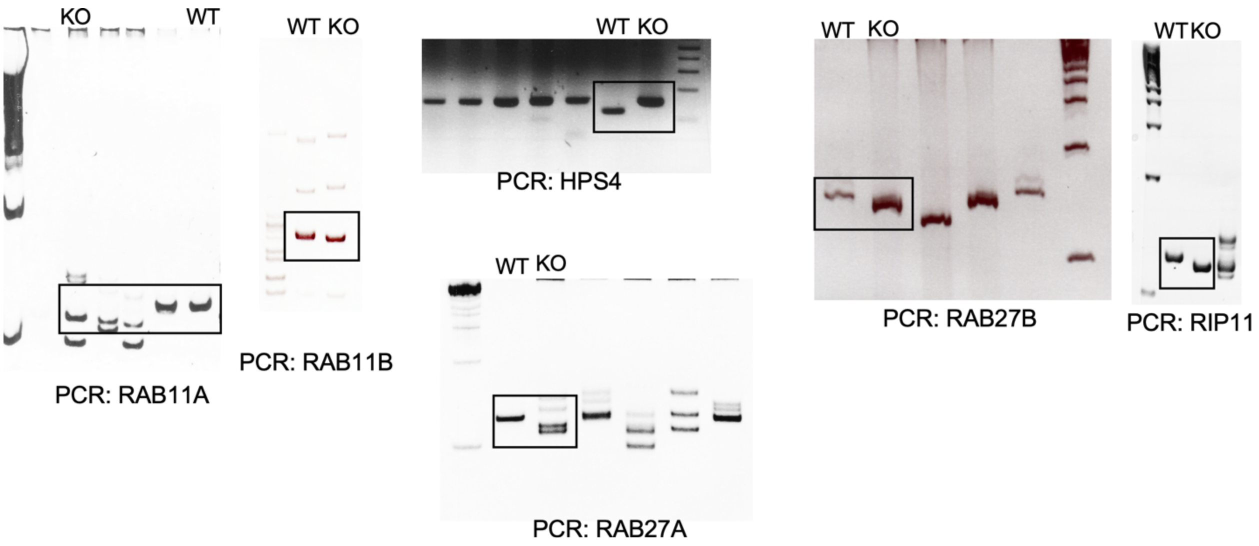

**Figure 5- source data 1**

**a**

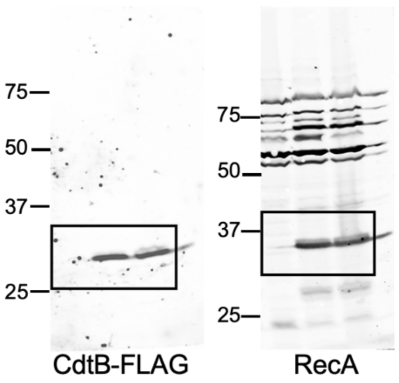

**Figure 5- source data 2**

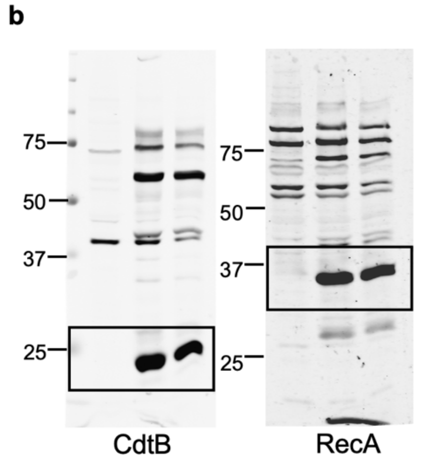

**Figure 6- figure supplement 6- source data 1**

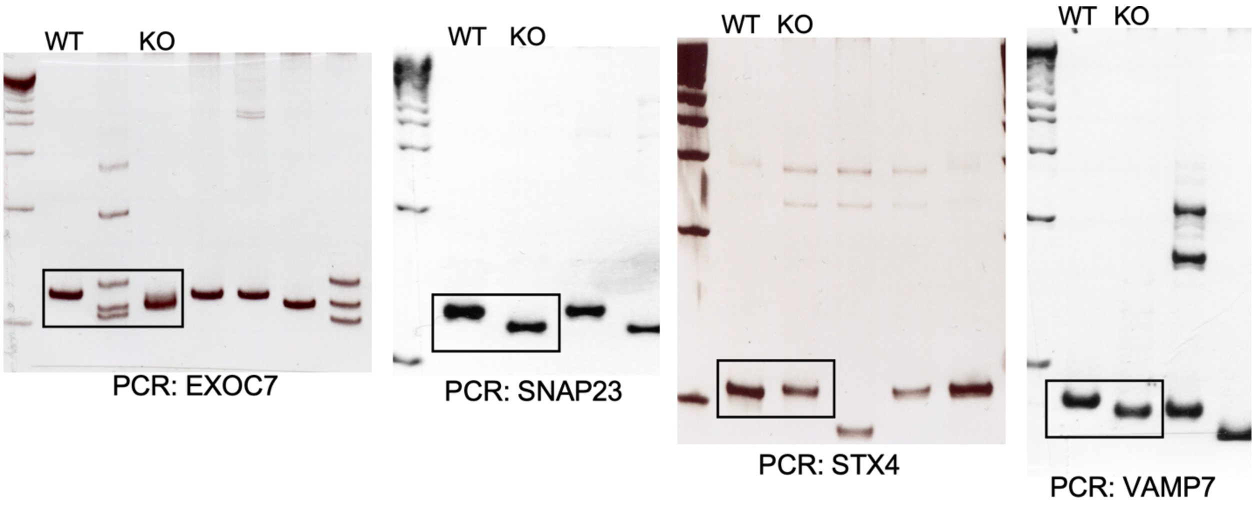

**Figure 6- source data 1**

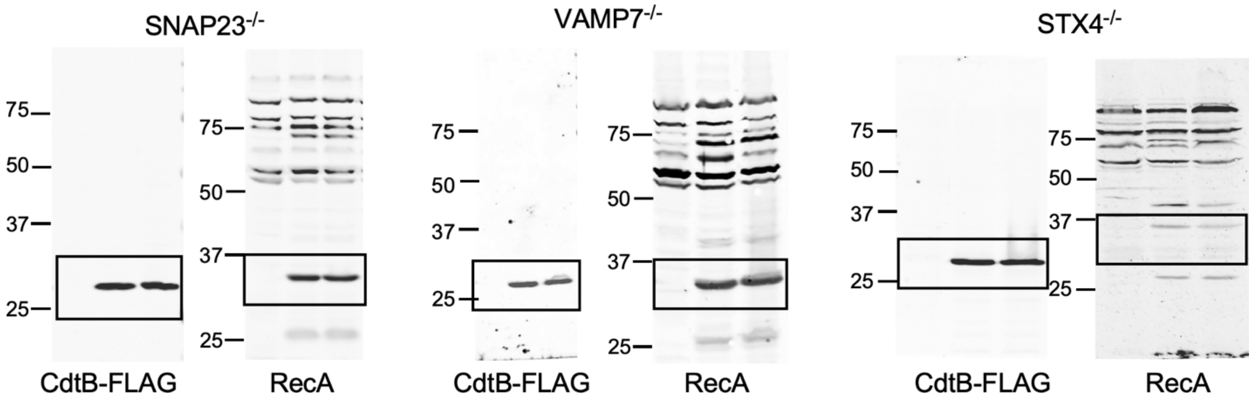

Supplement: Source data 1. [file elife-78561-data1.pdf]
